# Safety of Men With Small and Medium Abdominal Aortic Aneurysms Under Surveillance in the NAAASP

**DOI:** 10.1161/CIRCULATIONAHA.118.036966

**Published:** 2019-01-14

**Authors:** Clare Oliver-Williams, Michael J. Sweeting, Jo Jacomelli, Lisa Summers, Anne Stevenson, Tim Lees, Jonothan J. Earnshaw

**Affiliations:** 1 Cardiovascular Epidemiology Unit, Department of Public Health & Primary Care, University of Cambridge, UK (C.O.-W., M.S.).; 2 Homerton College, University of Cambridge, UK (C.O.-W.).; 3 Department of Health Sciences, University of Leicester, UK (M.S.).; 4 Public Health England, Vulcan House, Sheffield, UK (J.J., L.S., A.S.).; 5 University Hospitals, Newcastle, Newcastle upon Tyne, UK (T.L.). Gloucestershire Hospitals National Health Service Foundation Trust, Cheltenham, UK.

**Keywords:** aortic aneurysm, abdominal, epidemiology, mass screening, men, ultrasonography

## Abstract

**Background:**

Population screening for abdominal aortic aneurysm (AAA) has commenced in several countries, and has been shown to reduce AAA-related mortality by up to 50%. Most men who screen positive have an AAA <5.5 cm in diameter, the referral threshold for treatment, and are entered into an ultrasound surveillance program. This study aimed to determine the risk of ruptured AAA (rAAA) in men under surveillance.

**Methods:**

Men in the National Health Service AAA Screening Programme who initially had a small (3–4.4 cm) or medium (4.5–5.4 cm) AAA were followed up. The screening program’s database collected data on ultrasound AAA diameter measurements, dates of referral, and loss to follow-up. Local screening programs recorded adverse outcomes, including rAAA and death. Rupture and mortality rates were calculated by initial and final known AAA diameter.

**Results:**

A total of 18 652 men were included (50 103 person-years of surveillance). Thirty-one men had rAAA during surveillance, of whom 29 died. Some 952 men died of other causes during surveillance, mainly cardiovascular complications (26.3%) and cancer (31.2%). The overall mortality rate was 1.96% per annum, similar for men with small and medium AAAs. The rAAA risk was 0.03% per annum (95% CI, 0.02%–0.05%) for men with small AAAs and 0.28% (0.17%–0.44%) for medium AAAs. The rAAA risk for men with AAAs just below the referral threshold (5.0–5.4 cm) was 0.40% (0.22%–0.73%).

**Conclusions:**

The risk of rAAA under surveillance is <0.5% per annum, even just below the present referral threshold of 5.5 cm, and only 0.4% of men under surveillance are estimated to rupture before referral. It can be concluded that men with small and medium screen-detected AAAs are safe provided they are enrolled in an intensive surveillance program, and that there is no evidence that the current referral threshold of 5.5 cm should be changed.

**Clinical Perspective**

**What Is New?**

- There remains debate about when to intervene in patients with a large abdominal aortic aneurysm (AAA).
- This study concerns men aged >65 years with an AAA in surveillance detected either through population screening or self-referral.
- With the use of a standard surveillance schedule, the risk of rupture in AAA measured using the inner-to-inner ultrasound method was <0.5% per annum, even in men whose AAA was just <5.5 cm, the current referral threshold.

**What Are the Clinical Implications?**

- Current surveillance procedures in the National Health Service AAA Screening Programme result in very low rupture risks.
- Men with small and medium screen-detected AAAs are safe in an intensive surveillance program, and there is no evidence that the current referral threshold of 5.5 cm should be changed.

**Editorial, see p 1381**

Abdominal aortic aneurysm (AAA) ultrasound screening programs are emerging worldwide.^1,2^ The majority of programs are based on a single scan in elderly men, who are reassured and discharged if an AAA is excluded (usually if the abdominal aorta is <3.0 cm in diameter). A minority of screened individuals are diagnosed with large AAAs (usually >5.4 cm in diameter), and are referred to a vascular specialist for consideration of intervention. A further group has a small (3.0–4.4 cm) or medium (4.5–5.4 cm) AAA that is not immediately life-threatening, but warrants monitoring. These people are usually offered ultrasound surveillance with referral for treatment once the threshold for intervention (usually 5.5 cm diameter) is reached.

After a population-screening program has become established, a substantial number of men will be under ultrasound surveillance. It is important to be able to show that AAA surveillance schedules are safe, and that men with small and medium AAAs below the referral threshold are not at unacceptable risk of AAA rupture.

The National Health Service AAA Screening Programme (NAAASP) invites all men aged 65 years in England for an abdominal ultrasound scan. Men aged >65 years who have never been screened may self-refer.^3^ Men with small or medium AAAs are offered surveillance: annually for small AAAs, and every 3 months for medium AAAs. This schedule is based on evidence from the results of previous randomized trials of AAA screening and population programs.^4,5^ Details of the NAAASP standard operating procedure are recorded elsewhere.^6^ Some 300 000 men each year reach the age of 65 years and are invited for screening; in 2018, the 2 millionth man was invited.

The present threshold for referral for treatment of 5.5 cm in NAAASP is based on the Small Aneurysm^7^ and Aneurysm Detection and Management^8^ trials, where it was shown that it was as safe to be under surveillance as it was to have early intervention for men with an AAA <5.5 cm in diameter.

It is well known that men with small and medium AAAs have coexisting medical conditions. They are often smokers, and many have smoking-related conditions including cancer.^9,10^ Men with small and medium AAAs benefit from the best medical therapy: usually secondary prevention with antiplatelet medications and statins.^11^ These medications, along with lifestyle improvements and smoking cessation, are recommended for men under surveillance in NAAASP.

The main aim of the present study was to ensure the safety of men under surveillance in NAAASP. Because the aim of the program is to prevent death from AAA rupture, the study examined fatal and nonfatal AAA ruptures in men with small and medium AAAs in the surveillance program. The risk of rupture during surveillance should be substantially less than the risk of in-hospital death from elective AAA repair (1.4% for open and endovascular repair in the latest report from the UK National Vascular registry^12^). The secondary aim was to examine other causes of death in the surveillance cohort, with the long-term aim of designing medical interventions that might reduce the risk of any death in surveillance.

## Methods

The study was approved by the NAAASP Research Committee and performed as part of its program evaluation. Men who accepted the invitation and attended for ultrasound imaging gave verbal consent to screening and to having their anonymized information stored and used for program evaluation, which is recorded. The methods used for analysis and materials used to conduct the research are available to any researcher, but the original data are subject to the current rules of Information Governance.

After screening, which was done using the inner-to-inner ultrasound measurement method,^13^ men with small (3.0–4.4 cm) and medium (4.5–5.4 cm) AAAs detected in NAAASP were invited to join a surveillance program; those who agreed and attended were included in the analysis. Data concerning their attendance and outcomes were collected within the program on a bespoke information technology system, AAA SMaRT (Screening Management and Referral Tracker). All men were offered an appointment with a nurse specialist on entering surveillance, where baseline data were obtained and advice was given about a healthy lifestyle and best medical management. The management of men identified with an AAA after invitation at 65 years and of those who self-referred was identical.

Men were offered regular ultrasound scans using monitored standards. The reproducibility of the scan measurements has been demonstrated previously in this cohort.^13^ Reasons for leaving surveillance were recorded. Also recorded on the database were details of surveillance scans (dates, aortic diameter) and outcomes including death, referral for surgery, declines, and loss to follow-up. Dates and causes of death during surveillance, and details concerning any men who had a ruptured AAA but survived, were obtained from local screening programs as part of program monitoring. Local screening programs were advised to review death certificates, postmortem results, or hospital discharge summaries, or to contact treating clinicians or family doctors directly to determine cause of death as accurately as possible.

### Statistical Analysis

Data from AAA SMaRT were extracted up to August 18, 2017. The first men were included in surveillance once the program commenced in 2009. Follow-up was defined as from the time of first scan until death, referral to a vascular surgeon, rupture, loss to follow-up, or the administrative censoring date of August 18, 2017, whichever came first. Loss to follow-up was not always recorded, so patients were censored 18 months after the last scan if no further information was recorded.

Baseline characteristics and outcomes were tabulated by route of entry into the screening program: either routinely invited or self-referred. Continuous variables were presented as median and interquartile ranges, and categorical variables were presented as number and percentage.

All analyses included self-referred and routinely invited men and were restricted to events that occurred before referral for surgery.

The number of person-years of follow-up, AAA ruptures, deaths, and the mortality and rupture rates per 100 person-years were calculated by referral status (self-referred or routinely invited), and aortic diameter, stratified by small and medium AAA categories, and also by 3.0 to 4.9 cm and 5.0 to 5.4 cm categories. The latter group had AAAs just below the threshold for referral and might be considered to be at highest risk of rupture.

Analyses by aortic diameter were conducted using both the initial aortic diameter and a time-updated measurement (time-varying covariate). The time-updated measurement split the time that individuals contributed before their censoring date into the relevant aorta diameter categories, as determined by the previous measurements. For example, men who initially had an aneurysm of <4.5 cm, which increased to an aneurysm of >4.5 cm during follow-up, contributed time to both the small and medium AAA categories.

Age-standardized mortality ratios were calculated, stratified by 5-year age group, using sex-specific combined English and Welsh data from the Office of National Statistics from 2011 to 2013.^14^ The nonparametric cumulative incidence curve was calculated for the time to death under surveillance by initial aortic diameter categories, and 95% CIs were based on the log-log transformation of the cumulative incidence.^15^ Rupture and referral for consideration of intervention were treated as competing events in these analyses. Stacked cumulative incidence curves for each of the 3 competing events (rupture, referral for intervention, and death without rupture or referral) were produced to assess the total risk of each event during surveillance for all men combined. The incidence of each outcome was also estimated by initial aortic diameter (3.0–3.4 cm, 3.5–3.9 cm, 4.0–4.4 cm, 4.5–4.9 cm, and 5.0–5.4 cm), with stacked cumulative incidence curves produced separately for each category.

All analyses were conducted using STATA release 14 (Stata Corp).

## Results

Between 2009 and 2017, a total of 18 652 men were found to have an initial AAA of ≥3.0 cm in NAAASP. The majority of the men (83.2%) were routinely invited for screening (they did not self-refer). Self-referred men were, on average, older (75.4 versus 65.0 years, *P*<0.001), and more likely to be nonsmokers at nurse assessment (Table 1). There was no difference in the proportion of deaths, ruptures, or loss to follow-up between the 2 groups.

**Table 1.**
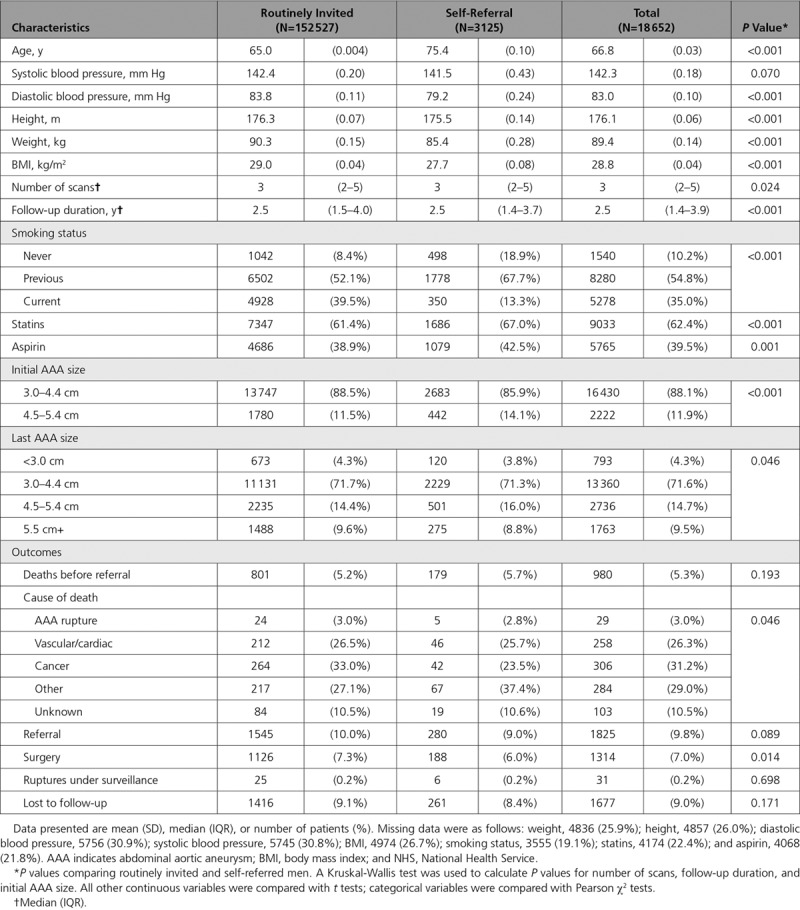
Characteristics of Participants With Initial Diameter ≥3.0 cm in the NHS Abdominal Aortic Aneurysm Screening Programme, 2009 to 2017

During follow-up, 1763 men had a scan measurement of ≥5.5 cm, of whom 1742 were referred to a vascular service for consideration of treatment. A further 83 men were referred before their AAA size reached 5.5 cm, usually because of an iliac aneurysm, tenderness, rapid aneurysm growth, or other finding. Of men whose aorta measured ≥5.5 cm, 94.9% were referred to the vascular service within 1 day, which is the program standard. Of the 21 men who were not referred, 16 declined referral, 2 left the surveillance program, 2 died of non-AAA causes, and 1 was referred after data collection ended on August 18, 2017.

### AAA Rupture

Thirty-one men had a ruptured AAA during surveillance: 25 routinely invited men and 6 self-referred men (Table 2). Twenty-nine (93.5%) of the men who had a ruptured AAA died. There was little difference in the incidence rate of rupture between routinely invited and self-referred men: 0.06 (95% CI, 0.04–0.09) and 0.08 (95% CI, 0.03–0.17) per 100 person-years, respectively (Table 2). Therefore, we combined the groups when calculating rupture rates by aortic diameter categories.

**Table 2.**
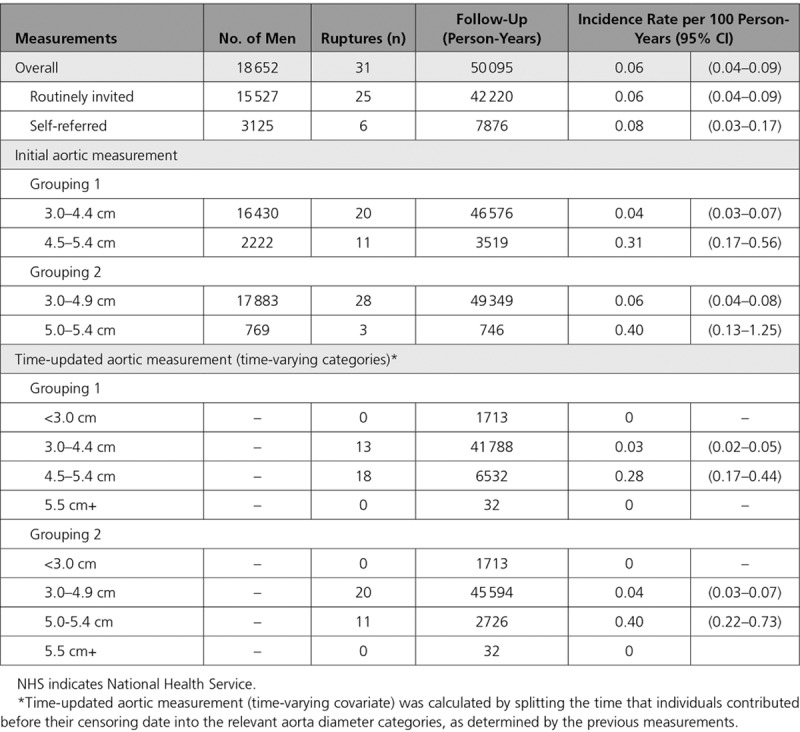
Rates of Ruptures That Occurred in the NHS Abdominal Aortic Aneurysm Screening Programme During Surveillance, 2009 to 2017

**Figure 1.**
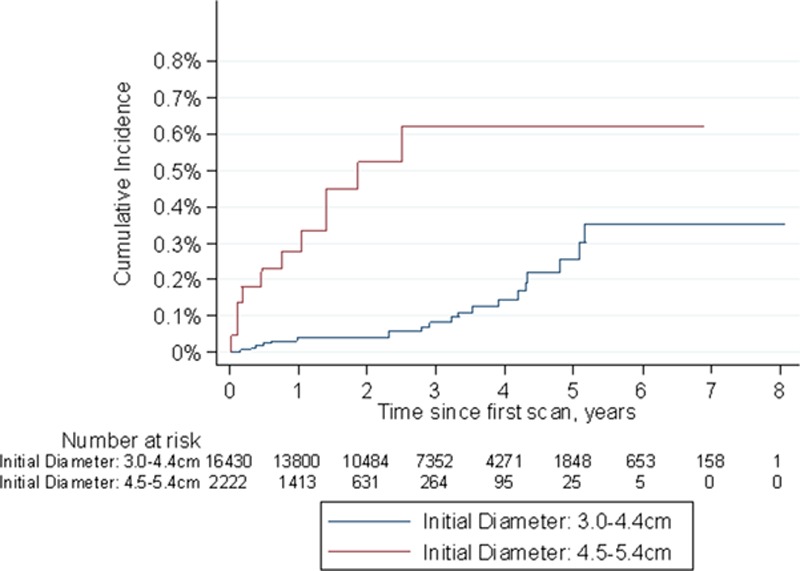
**Cumulative incidence of rupture during surveillance in the National Health Service Abdominal Aortic Aneurysm Screening Programme, 2009 to 2017, by initial diameter (3.0–4.4 cm/4.5–5.4 cm).** Incidence of rupture was estimated with referral and mortality as competing risks.

The estimated rupture rate was <1 per 100 person-years for all diameter categories, but was highest for men with an aortic measurement of 5.0 to 5.4 cm: 0.40 (95% CI, 0.13–1.25) per 100 person-years based on initial aortic measurement, and 0.40 (95% CI, 0.22–0.73) per 100 person-years based on time-updated aortic measurements (Table 2). Using time-updated measurements, there was strong evidence that the rupture rate was <1 per 100 person-years for diameters 5.0 to 5.4 cm (*P*<0.001). The cumulative incidence of rupture during surveillance reached 0.62% in men with medium aneurysms at baseline and 0.35% for men with small aneurysms at baseline (Figure 1). Overall, an estimated 0.4% of men are assumed to rupture while under surveillance.

### Mortality

Of the initial cohort of 18 652 men, 980 (5.3%) died in surveillance, of which 29 (3.0%) died of ruptured AAAs (Table 1). Other deaths were attributable to cancer (31.2%), vascular or cardiac causes (26.3%), and noncardiovascular and noncancer causes (29.0%). The causes of death for 103 men (10.5%) were unknown. Individuals whose initial AAA diameter was in the range 4.5 to 5.4 cm had a higher proportion of deaths from AAA rupture than individuals whose initial AAA diameter was 3.0 to 4.4 cm (14.5% versus 2.1%).

**Figure 2.**
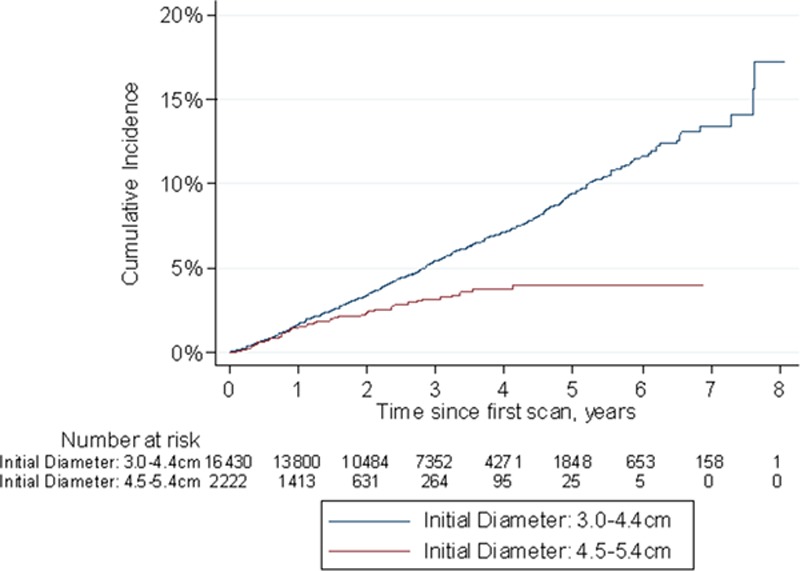
**Cumulative incidence of death during surveillance in the National Health Service Abdominal Aortic Aneurysm Screening Programme, 2009 to 2017, by initial diameter (3.0–4.4 cm/4.5–5.4 cm).** Incidence of death was estimated with referral and mortality as competing risks.

There were an estimated 1.96 deaths per 100 person-years (95% CI, 1.84–2.08) in men under surveillance before referral (Table 3). The mortality rate was similar for men who initially had a medium aneurysm. When based on time-updated aortic measurements, mortality rates increased with AAA size, with the smallest rates for men with AAA of <3.0 cm, and greatest for those with AAA of ≥5.5 cm. A similar cumulative incidence of death was estimated for men with small and medium aneurysms within the first year of surveillance: 1.66% (95% CI, 1.47%–1.87%) and 1.46% (1.01%–2.05%), respectively (Figure 2). By the third year, an estimated 5.42% (5.02%–5.84%) of men who initially had a small aneurysm and 3.16% (2.37–4.12%) of men with a medium aneurysm had died while in surveillance. The lower incidence for men with a medium aneurysm is attributable to accounting for the greater referral rates in this group.

**Table 3.**
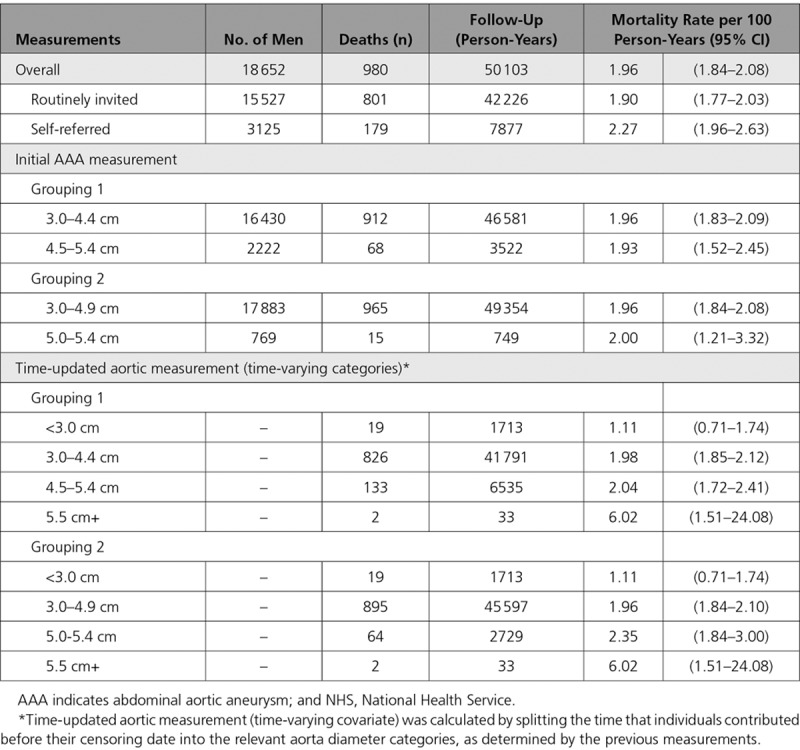
Mortality Rates During Surveillance in the NHS Abdominal Aortic Aneurysm Screening Programme, 2009 to 2017, by Initial Aortic Measurement and Last Known Aortic Measurement

The mortality rate increased with age for both routinely invited (Table 4) and self-referred men (Table 5). For routinely invited men, the mortality rate was ≈60% higher than in the male, age-matched English and Welsh population, but for self-referred men it was ≈40% lower. However, because self-referred men were on average older, this led to overall mortality rates similar to routinely invited men.

**Table 4.**
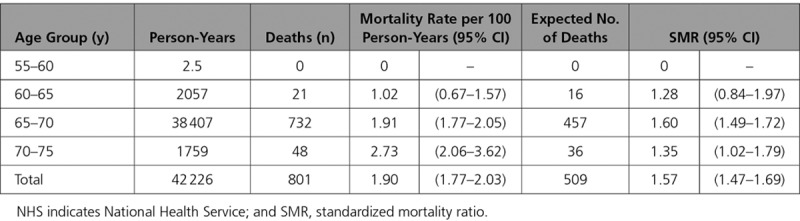
Standardized Mortality Ratios for Routinely Invited Men While Under Surveillance in the NHS Abdominal Aortic Aneurysm Screening Programme, 2009 to 2017, Stratified by Age

**Table 5.**
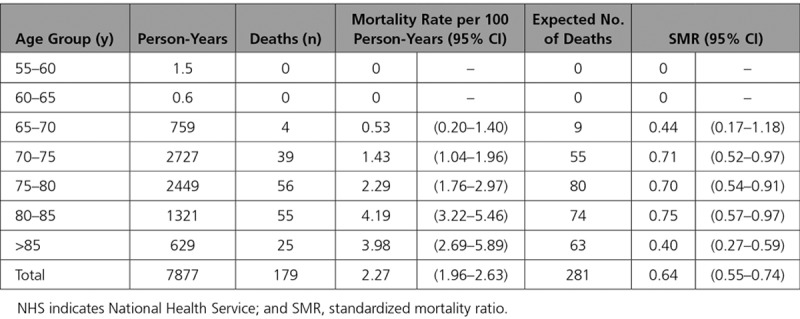
Standardized Mortality Ratios for Self-Referred Men While Under Surveillance in the NHS Abdominal Aortic Aneurysm Screening Programme, 2009 to 2017, Stratified by Age

**Figure 3.**
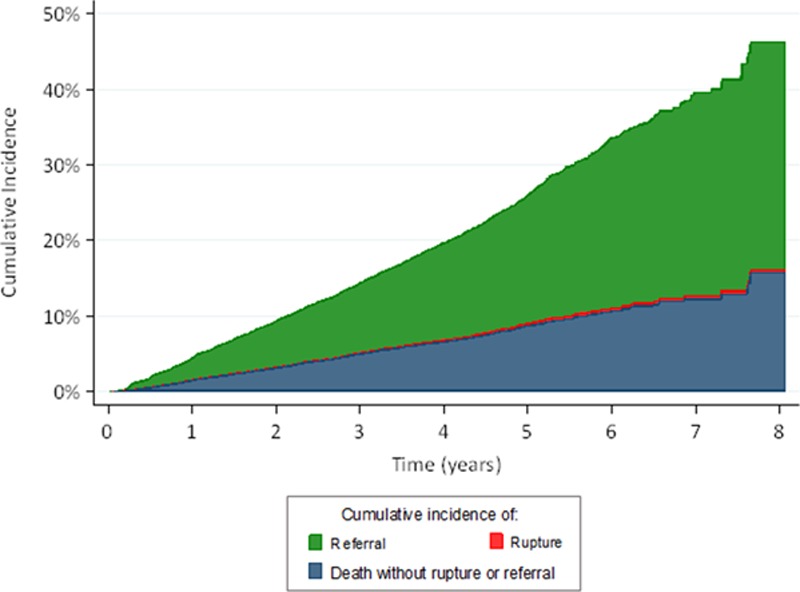
**Stacked cumulative incidence function plot for men under surveillance in the National Health Service Abdominal Aortic Aneurysm Screening Programme, 2009 to 2017.**

Figure 3 shows the cumulative incidence functions for each competing outcome (rupture, referral, and death without rupture) stacked on top of each other. The estimated probability of each outcome increases over the 8-year period of follow-up, with referral the most prevalent outcome. Approximately half of the population is estimated to remain under surveillance after 8 years.

**Figure 4.**
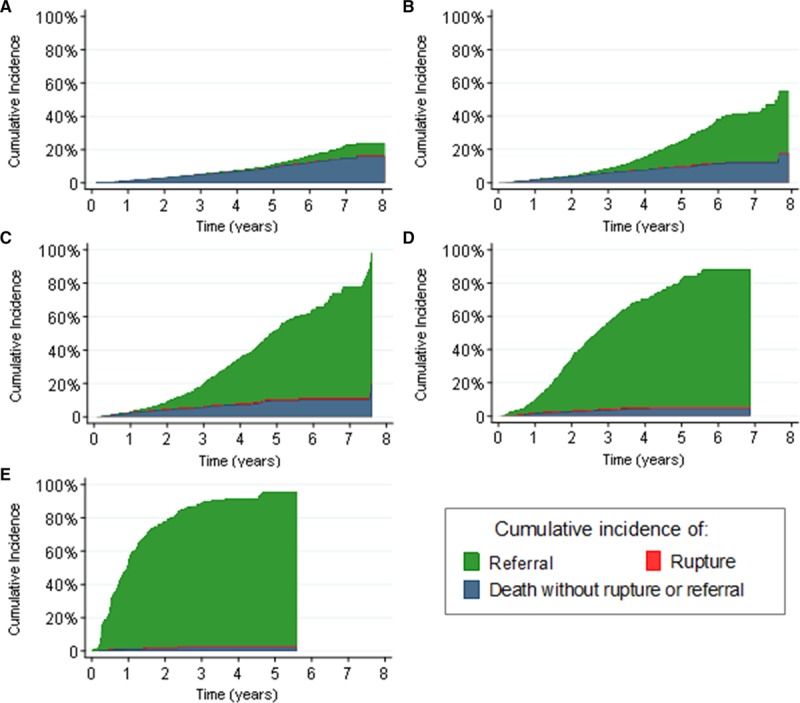
**Stacked cumulative incidence function plots for men under surveillance in the National Health Service Abdominal Aortic Aneurysm Screening Programme, 2009 to 2017, by initial diameter.** **A**, 3.0 to 3.4 cm. **B**, 3.5 to 3.9 cm. **C**, 4.0 to 4.4 cm. **D**, 4.5 to 4.9 cm. **E**, 5.0 to 5.4 cm.

Figure 4 displays the cumulative incidence of the same outcomes by initial aortic diameter. The estimated probability of referral within 8 years is high for men with initial diameters ≥4.0 cm, whereas the probability of rupture remains low for all diameter groups.

## Discussion

This study has shown that the suggested referral threshold of 5.5 cm measured by ultrasound imaging using the inner-to-inner method results in AAA rupture rates consistently <0.5% in men in surveillance. The present UK in-hospital mortality rate for elective AAA repair is ≈1.4%.^12^ After 8 years of surveillance, ≈50% of men had died or were referred for surgery. The cumulative incidence of rupture over 8 years was very low (0.4%). Therefore, it is considered that men enrolled in an intensive surveillance program such as NAAASP are safe, and that there is no evidence that the current NAAASP referral threshold of 5.5 cm should be changed. The risk of rupture is related to aortic diameter, but even in men with an AAA 5.0 to 5.4 cm, just below the threshold for referral, the rate was <0.5% per annum. This study examined only men with screen-detected AAAs, but it would be expected these findings would be generalizable to men under surveillance with small and medium AAA detected incidentally.

A small number of men under surveillance do rupture their AAAs, including small AAAs of <4.5 cm diameter. Some of these men may have developed an infection in their AAA (mycotic aneurysm) to account for early rupture. The majority of men who experience a rupture do not survive, despite knowledge of their condition. It had been thought that men known to have an AAA might be treated quicker if their AAA ruptures, but this is not supported by the present report.

The second conclusion is that the overall mortality rate in men under surveillance is higher than age-matched men in the general population, but only in the invited cohort. Self-referred men have a lower mortality. They have a lower body mass index and are less likely to be smokers, suggesting they are health-conscious, and possibly already medically well managed. Men with an AAA are known to have a higher risk of cardiovascular disease and to be at risk of premature death, mainly from smoking-related conditions.^10^ It has been shown previously that the risk of death from any cause is associated with increasing AAA diameter.^16^ The main causes of death in the men reported here were cardiovascular disease and cancer. Men in NAAASP are given advice about their health, in particular, smoking cessation, and the prescription of antiplatelets and statins is recommended. It has been shown that compliance with these medications improves the chances of survival.^11^ Optimizing the medical management of these men, possibly with regular monitoring, offers the best chance of reducing mortality in this cohort. The reduced mortality rate in self-referred men supports the expectation that good medical care can reduce mortality.

This study has a number of implications for practice. Patient referral thresholds for treatment of AAAs are largely derived from randomized controlled trials done in the 1990s.^7,8^ There has been argument that a single referral threshold for all people with an AAA is dated in the current era of personalized medicine. Scoring systems have been developed to identify subjects with an AAA for whom operation below the current threshold may be safe and improve cost efficacy.^17^ In Sweden, the perioperative risk of death after elective endovascular repair for AAA is 0.3%.^1^ It might be argued that, where intervention risks are small, a lower referral threshold is appropriate. Yet controlled trials, such as the CAESAR study (Comparison of Surveillance Versus Aortic Endografting for Small Aneurysm Repair) that compared early endovascular treatment with conservative treatment of small AAAs failed to show the superiority of early intervention.^18^ An alternative use of personalized scoring systems might be to delay intervention in men with a 5.5-cm AAA at low risk of rupture. There are a number of new biological and radiological imaging methods that could be used in this way.^19,20^

In addition, it is unlikely that the risk of AAA rupture suddenly becomes acute once an AAA reaches 5.5 cm in diameter. A controlled trial, randomly assigning men with AAAs 5.5 to 6.5 cm to treatment or continued surveillance, was never done because of the lack of equipoise among vascular surgeons. The positive effects of elective AAA repair are a balance of risk against benefit, accounting for postoperative quality of life, and life expectancy, as well; the balance for a fit 65-year-old man with a 5.5-cm AAA is clearly different from that of an 85-year-old man with the same sized AAA.^21^

The method used to assess aortic diameter in NAAASP is the inner-to-inner ultrasound method,^13^ which excludes the thickness of the aortic wall and usually measures ≈0.5 cm less than an aortic diameter measured on computed tomography. It could be argued that men in the NAAASP surveillance program may well be safe up to a diameter of 6.0 cm on computed tomography. This is a direct challenge to the use of 5.5 cm diameter on computed tomography as the threshold for intervention; thresholds should be different for each imaging modality. It is also in direct contrast to a recent publication criticizing surgeons in the United Kingdom for having a higher threshold for intervention than surgeons in the United States.^22^ This difference in threshold was used as an explanation for death from ruptured AAA being more common in the United Kingdom. The present results suggest that the explanation for this variation could be more complex.

The main limitation of this study is the fact that some of the causes of death were unknown. It is possible that some were attributable to ruptured AAA. The local screening programs have close ties with the vascular centers, and record any patient admitted with a ruptured AAA, so that information can be linked back. It is possible that some men who die suddenly at home, and do not have a postmortem, could have died of ruptured AAA. The study also concerns only men with AAA in surveillance. Population screening for AAA in women is not cost-effective,^23^ yet there will be women with small and medium AAAs who need surveillance. It is probably not appropriate to extrapolate the results of the present study to women, because there is some evidence that the referral threshold should be lower.^24,25^ Similarly, the findings may only be generalizable among populations with a similar ethnic composition such as England.^26^ The prevalence of AAA is known to vary among ethnic groups, although little is known about growth rate and rupture risks in various ethnicities.

The NAAASP has now been implemented for 5 years, and its outcomes are starting to be recorded.^2,26^ The present study is reassuring for current surveillance standards. It is possible that, with more data on men just below the threshold for referral, surveillance intervals could be relaxed, as recommended.^4^ It will take a decade before it will be possible to be sure to what extent the introduction of screening programs has had on the rate of AAA-related deaths. Although preliminary data from Sweden on the effectiveness of screening are conflicting,^3,27^ early signs are more encouraging in England.^27^

## Acknowledgments

The authors thank the staff and patients of the National Health Service AAA Screening Programme. All authors planned the study. Dr Jacomelli managed the National Health Service AAA Screening Programme information technology system and extracted the data. Dr Sweeting developed the analytic plan. Dr Oliver-Williams conducted the analyses and prepared the figures and tables with input from Dr Sweeting. Drs Oliver-Williams, Earnshaw, and Sweeting cowrote the first draft of the manuscript. All other authors contributed to the review of the final manuscript.

## Sources of Funding

This work was funded by the British Heart Foundation (RE/13/6/30180), and Homerton College, University of Cambridge. The National Health Service AAA Screening Programme, as part of Public Health England, is funded by the Department of Health.

## Disclosures

None.
